# The Victoria Hospital, Blackpool

**Published:** 1897-09-04

**Authors:** 


### THE VICTORIA HOSPITAL, BLACKPOOL.

### OPENING OF TWO NEW WARDS.

Sir Matthew White Ridley, M.P., the Home Secretary, opened the new wards of this hospital during the second week in August. He was received at the hospital by the matron, Miss Peel, and a large number of friends were present. Tea was served on the lawn, and then the company gathered in the south ward for the ceremony. Dr. Kingsbury introduced Sir Matthew Ridley, and presented him with a key. On this occasion the gift was amusingly symbolic, as the architect had forgotten to have the key-hole made, consequently it was impossible to turn the key in the lock. The new wards cost £4,000. The chairman defended the apparent excessive cost on the ground that everything is of the best. The floors are laid to secure durability, and, as far as possible, immunity from infection. The walls are partly adamantine and partly tile, the windows are double, and the heating is by stoves in the middle of the wards, and by hot pipes. The ventilation has been carefully considered. Sir Matthew, in declaring the wards open, stated that he had been authorised by Her Majesty to name the hospital the Victoria Hospital.

